# Extrapleural Intrathoracic Hydatid Cyst: A Rare Cause of Upper Limb Neuropathic Pain

**DOI:** 10.7759/cureus.51100

**Published:** 2023-12-26

**Authors:** Gaind Saurabh, Nirupam Chakraborty, Sneha Ghosh, Nitin Kumar Kashyap, Pranay Mehsare

**Affiliations:** 1 Cardiothoracic Surgery, All India Institute of Medical Sciences, Raipur, Raipur, IND; 2 General Surgery, All India Institute of Medical Sciences, Raipur, Raipur, IND

**Keywords:** extrapleural hydatid cyst, neuropathic pain, hydatid disease, echinococcosis, zoonotic disease

## Abstract

Human echinococcosis is a common zoonotic disease. Due to favourable climatic conditions, India contributes to the majority of the burden of cystic echinococcosis (CE) in the world. The lung is the most commonly affected organ in the body, after the liver. Common symptoms of pulmonary hydatid cyst (PHC) include cough, chest pain, expectoration, and hemoptysis. This case report is a rare presentation of hydatid disease of the thoracic cavity with complaints of upper limb neuropathic pain. Radiological investigation showed an extrapleural thoracic cyst compressing the brachial plexus, and serological findings confirmed the diagnosis of a hydatid cyst. The patient was taken up for cyst excision as it is the treatment of choice along with adjuvant chemotherapy.

## Introduction

Human echinococcosis is a zoonotic disease that is caused by tapeworms of the genus *Echinococcus*. Echinococcosis occurs in four forms: (a) cystic echinococcosis (CE), (b) alveolar echinococcosis, (c) two forms of neotropical echinococcosis, and (d) unicystic [1]. Cystic echinococcosis is the most common human disease of this genus, and it accounts for >95% of the estimated 2-3 million cases worldwide [2]. The hydatid tapeworm (*Echinococcus granulosus* (*E. granulosus*)) requires two hosts to complete its life cycle. Dogs are the definitive host; a variety of species of warm-blooded vertebrates like sheep, cattle, etc. are the intermediate hosts; and humans are the accidental hosts and do not take part in its biological cycle [3]. Cystic echinococcosis is an endemic zoonosis in many parts of the world, including India. The majority of the global human burden of CE is contributed by India, which is approximately 12% [4]. The lung is considered to be the second most infected part of the body in hydatid disease [5-7].

A pulmonary hydatid cyst (PHC) most commonly produces symptoms of cough followed by chest pain, breathlessness, expectoration, fever, hemoptysis, and anaphylactic phenomena [8]. We report a case of an unusual presentation of thoracic hydatid disease presenting with upper limb neuropathic pain.

## Case presentation

A 40-year-old female, a homemaker, presented with complaints of pain in her left upper limb for one and a half years. It was gradual in onset, originated from her left shoulder, and radiated until her fingertips. The pain was of the pins and needles type, which aggravated while working and was relieved by taking rest and medications. The pain gradually increased in intensity and was not relieved by medication. The pain was also associated with swelling on the left side of the base of the neck, which gradually increased in size. There were no complaints of chest pain, cough, or breathlessness. On examination, she was haemodynamically stable. The neurological examination revealed that, apart from the pins and needle sensation in the left upper limb, there was no other sensory or motor neurological deficit. The respiratory system examination showed no obvious abnormality.

**Figure 1:**
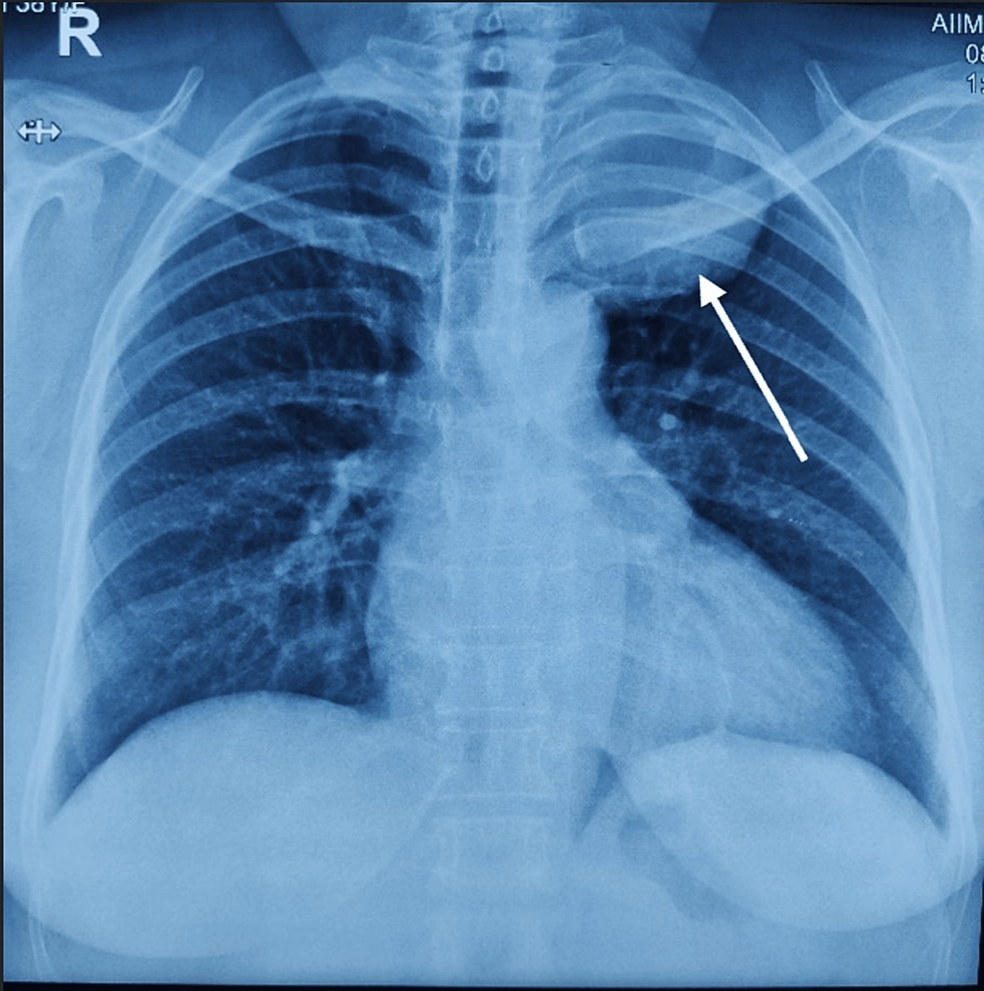
Chest X-ray (posteroanterior (PA) view) showing a spherical homogenous opacity at the apex (pointed with the white arrow).

The chest X-ray revealed a circular homogenous opacity located at the apex of the left thoracic cavity with smooth, regular borders pushing the left lung upper lobe; superior margins could not be traced completely (Figure 1).

**Figure 2:**
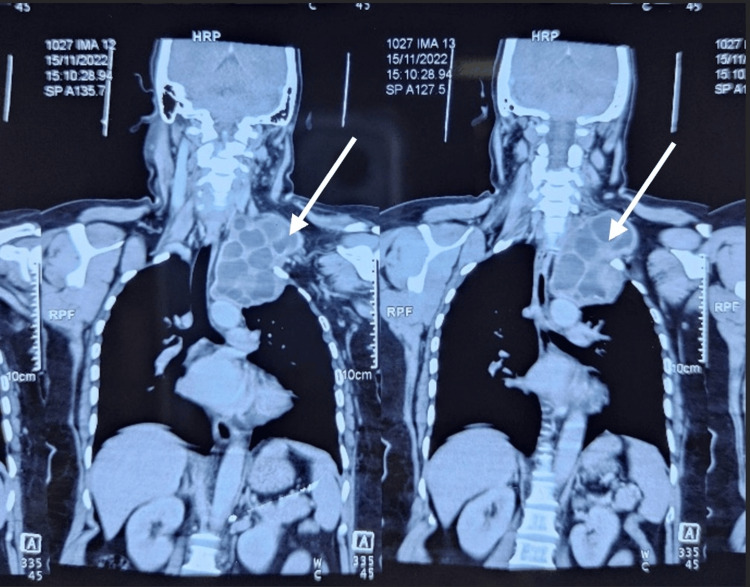
A CT scan of the thorax (coronal section) showing the multiloculated cystic lesion (pointed with the white arrow) extending through the thoracic inlet.

**Figure 3:**
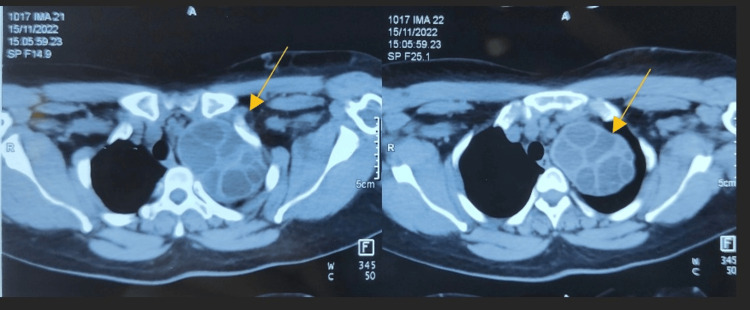
A CT scan of the thorax (cross-section) showing the multiloculated cystic lesion (pointed with the yellow arrow) extending through the thoracic inlet.

Ultrasonography of the neck on the left side showed a large multiloculated cystic lesion, the caudal extension of which could not be traced. A USG-guided aspiration of the cystic lesion showed a clear, watery aspirate. On further radiological investigations, a CT scan of the thorax showed a well-defined extrapleural multiloculated cystic lesion measuring around 6.3 x 7.7 x 8.8 cm in the upper aspect of the left thoracic cavity and seen extending through the thoracic inlet to displace the left carotid vessels anteriorly and compress the left subclavian artery and vein (Figures 2-3).

**Figure 4:**
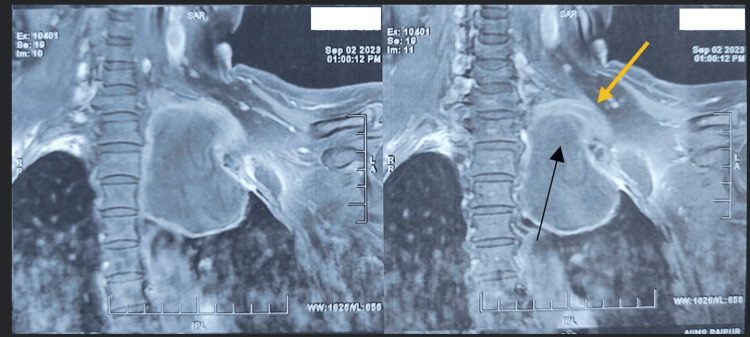
An MRI of the brachial plexus demonstrating the cyst (pointed with the black arrow) abutting the left brachial plexus (pointed with the yellow arrow).

An MRI of the brachial plexus showed a large, well-defined, round, multiloculated, septate cystic mass involving the upper lobe of the left lung with extrapulmonary extension into the left pre-cervical space region extending anteriorly from C6 to D4 vertebral body levels, and lesions involving the trunks and divisions of the C7 to D1 nerves-features suggestive of a hydatid cyst (Figure 4).

A USG of the abdomen was found to be within normal limits. All the haematological investigations were within normal limits except for serology for *Echinococcus *IgG (20.94), which was found to be elevated. With the combination of radiological and serological evidence, the diagnosis was confirmed to be a hydatid disease of the lung.

**Figure 5:**
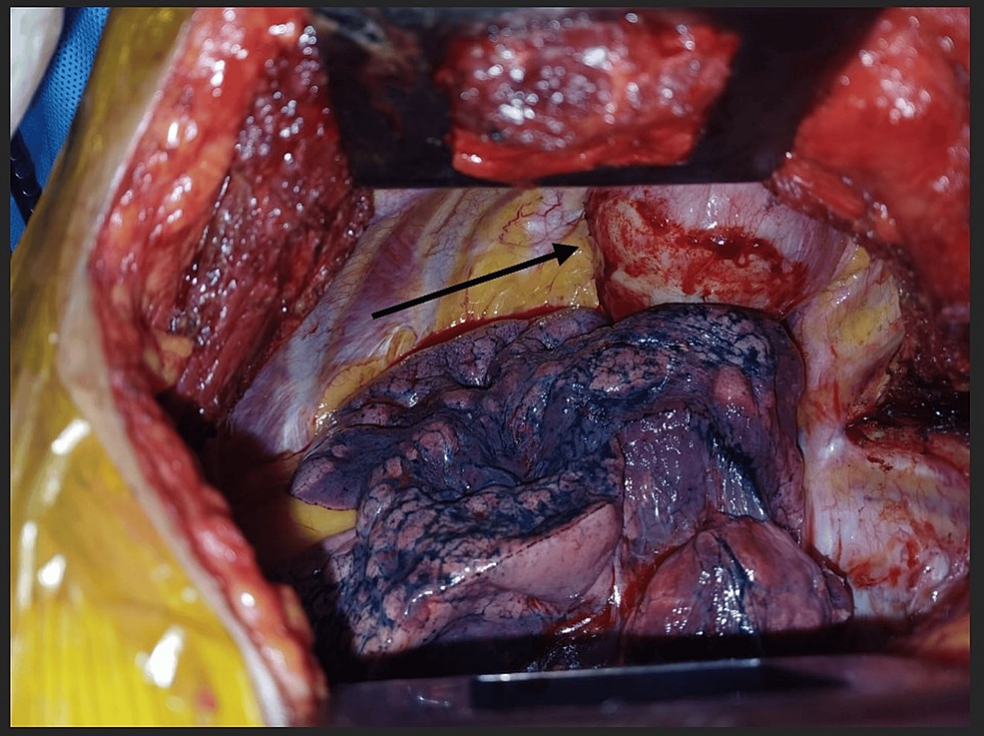
Intraoperative finding depicting a large 6 x 6 cm cyst in the upper part of the thoracic cavity (pointed with the black arrow).

Due to some financial constraints from the patient, she was started on tab. albendazole 400mg per oral (PO) twice daily (BD) for three months prior to planned surgery. A repeat CT scan of the thorax showed the presence of two hydatid cysts, one as described above with minimal reduction in size (6.3 x 7.7 x 6.8 cm) and another along the superior segment of the left lower lobe. The patient was taken up for surgical excision of the hydatid cyst. Under general anaesthesia, the thoracic cavity was exposed via left posterolateral thoracotomy. On exploration, a large 6 x 6 cm hydatid cyst was found in the upper part of the thoracic cavity, just abutting the upper lobe with fine adhesions, and part of it was going through the thoracic inlet into the neck (Figure 5).

**Figure 6:**
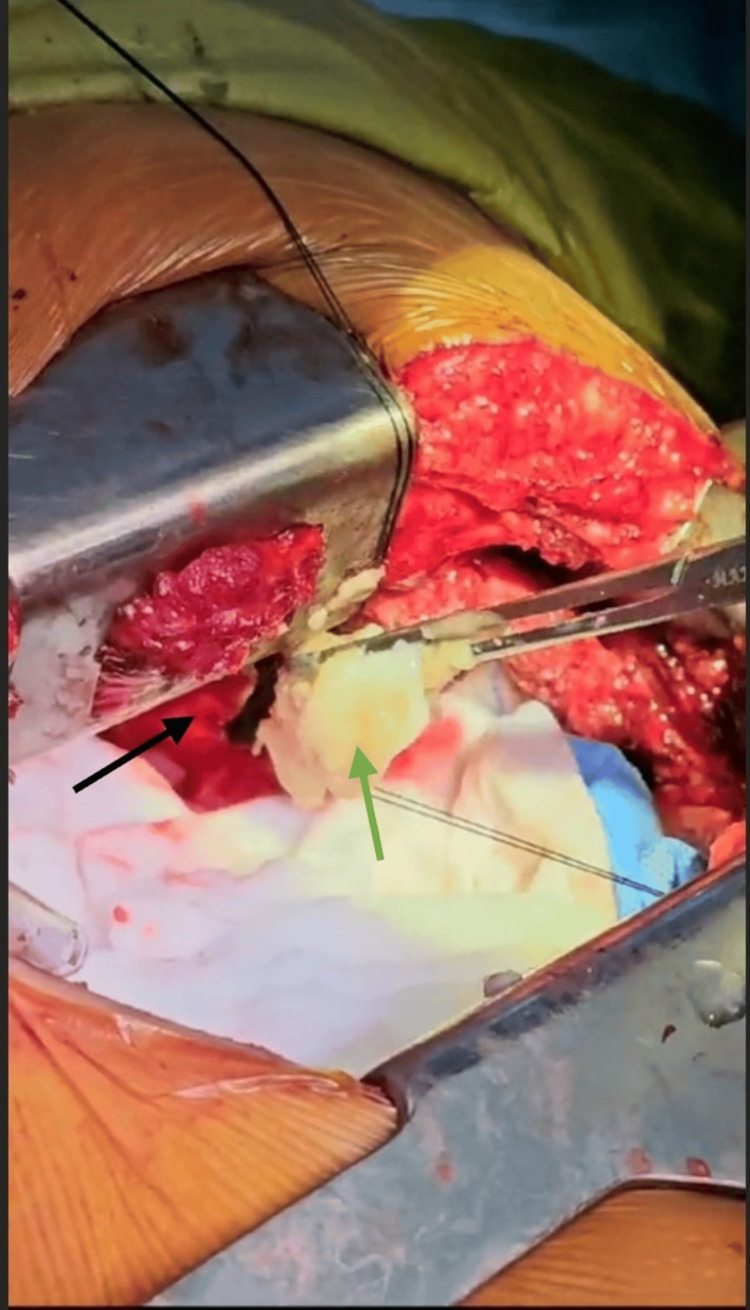
Decompression of the cysts by the removal of the daughter cysts. Pericyst (pointed with a black arrow) and evacuated daughter cysts (pointed with a green arrow) are visualized.

The upper lobe was separated by adhesiolysis, and the cyst was seen, densely adhered in and around the thoracic inlet. Thus, hydatid cyst enucleation was done so as not to inflict any injury to the brachial plexus, and a thorough lavage was given with mannitol, 10% povidone-iodine, and hydrogen peroxide, leaving behind the pericyst (Figure 6).

A small 2 x 2 cm hydatid cyst was noted in the superior aspect of the left lower lobe, which was excised and capitonage done. Histopathological examination of the cyst wall confirmed it to be a hydatid cyst. Postoperatively, the patient had a significant reduction in the pain of the left upper limb and was discharged on postoperative prophylaxis of tab. albendazole 400mg PO BD for three months to prevent recurrence.

## Discussion

This case report illustrates one of the unusual presentations of PHC caused by a mass effect. Echinococcosis is an endemic zoonotic disease in India responsible for a major public health burden. Around 12% of the global CE cases are from India, which is because of favourable climatic conditions, the availability of large numbers of intermediate hosts dependent on grazing activities, a lack of awareness, and poor collaboration between the veterinary and medical sciences in India [2].

The initial phase of primary infection is asymptomatic and may remain so for many years. Hydatid disease is seen in subjects of any age and sex, although it is more common in those aged between 20 and 40 years [9-10]. Once the conditions are favourable for the development of the parasite, a hydatid cyst develops, which consists of three layers: (a) the outer pericyst caused by the host’s inflammatory reaction; (b) the middle laminated membrane; and (c) the endocyst, which is the germinative layer. The size of the hydatid cyst may vary from a few centimetres to very large cysts. Due to the prolonged asymptomatic course of the disease, many patients present with complaints due to mass effects or rupture of the cyst.

The diagnosis of hydatid disease is done with a combination of radiological and serological tests. Although chest radiography is the primary diagnostic method, CT and MRI are used to delineate the exact extensions of the lesion. Apart from demonstrating the lesions, they are also helpful in looking for treatment responses and predicting complications. Though a non-contrast CT scan can demonstrate hydatid cysts, a contrast-enhanced CT scan of the thorax is required to look for other lesions, especially those located in the mediastinum [11]. Among the patients with lung cysts, 20%-40% also have liver cysts [3]. Therefore, a USG of the whole abdomen is performed to rule out the concurrent presence of a liver hydatid cyst.

Surgical excision is the treatment of choice in thoracic hydatid disease. The most common procedure for the management of lung hydatid cysts is Barrett/Posadas’ technique (cystotomy and closure of bronchopleural fistulas with or without capitonnage) [12].

In the above-presented case, the patient had major relief in the left upper limb radiating pain and was kept on chemoprophylaxis for three months post-surgery with tab. albendazole 400mg PO BD to prevent the recurrence and also on neuroprotective agents like methylcobalamin for the residual mild pain.

## Conclusions

Hydatid disease is a common zoonotic disease in tropical countries like India. There can be various presentations of pulmonary hydatid cysts, which include chest pain and cough. Compressive neuropathic pain is a very rare and unusual presentation of a PHC, which we must be aware of, and hence, it has been discussed in this report. A diagnosis of a hydatid disease can be kept as a differential diagnosis for unexplained neuropathic pain.
